# Case report: Be alert to parvovirus infection in patients with unexplained anemia after cerebral hemorrhage surgery

**DOI:** 10.3389/fmed.2026.1700344

**Published:** 2026-02-19

**Authors:** Muhua Dai, Lisha Pang, Mahong Hu, Jianbiao Meng, Chunlian Ji, Lingxiang Sheng, Wei Zhang

**Affiliations:** Department of Critical Care Medicine, Tongde Hospital of Zhejiang Province Afflicted to Zhejiang Chinese Medical University, Hangzhou, Zhejiang, China

**Keywords:** human parvovirus B19, mNGS, organ failure, PVB19, refractory anemia

## Abstract

**Background:**

Human parvovirus B19 (PVB19) is a highly prevalent single-stranded DNA virus that infects a large proportion of the global population. It can involve multiple organ systems, leading to a broad spectrum of clinical manifestations. While most infections in immunocompetent individuals are mild and self-limiting, PVB19 can occasionally cause severe and diverse complications.

**Case presentation:**

We report a rare case of an immunocompetent patient who experienced unexplained clinical deterioration following surgical evacuation of an intracerebral hemorrhage. The patient presented with refractory anemia, impaired consciousness, fever, seizures, and progressive dysfunction of the cardiac, hepatic, and renal systems. Metagenomic next-generation sequencing revealed high levels of PVB19 DNA in the cerebrospinal fluid, blood, and pleural effusion. The patient was treated with intravenous immunoglobulin (IVIG) therapy and supportive care. Following treatment, improvements were observed in consciousness, mobility, and anemia. However, renal function failed to recover and ultimately progressed to renal failure, necessitating renal replacement therapy.

**Conclusion:**

This case underscores the potential severity of PVB19 infection following cerebral hemorrhage surgery, particularly when accompanied by unexplained anemia. Accurate diagnosis requires a high index of suspicion and the use of advanced diagnostic tools. Management primarily involves IVIG therapy and supportive care. This case highlights the importance of expanding the differential diagnosis in postoperative patients presenting with unexplained anemia and multi-organ dysfunction, as early recognition of atypical infections may improve clinical outcomes.

## Introduction

PVB19 is a single-stranded DNA virus first identified accidentally in 1975 by Australian virologist Yvonne Cossart (1). Parvovirus B19 infection is common, since the seroprevalence of PVB19 exceeds 76% in adults worldwide (2). The clinical manifestations of PVB19 infection vary widely in severity, depending largely on the patient’s immune status and hematological status. In immunocompetent individuals (3), PVB19 infection typically causes erythema infectiosum a mild rash illness primarily affecting children and may lead to polyarthritis or arthralgia in adults. In pregnant women, infection can result in fetal hydrops, intrauterine fetal death, or miscarriage (4). Among immunocompromised patients, PVB19 is associated with pure red cell aplasia (PRCA) and transient aplastic crisis (TAC) (5, 6). Fetal cerebellar hemorrhage has been reported in fetuses following PVB19-induced severe anemia and subsequent intrauterine transfusion (7). Recent studies have broadened the recognized clinical spectrum of PVB19, linking it to systemic sclerosis, hepatitis, myocarditis, encephalitis, meningitis, hemophagocytic lymphohistiocytosis, and renal impairment (8–10). The underlying pathogenic mechanisms remain incompletely understood but are thought to be primarily immune-mediated (8, 11, 12). Currently, no specific antiviral therapy exists; treatment is largely supportive, focusing on symptom control and organ function preservation. In severe cases, IVIG therapy or blood transfusion may be required.

Reports of multiple organ dysfunction or failure caused by PVB19 infection in previously immunocompetent individuals are rare. To date, no cases have been documented in postoperative intracerebral hemorrhage patients developing multiple organ failure secondary to PVB19 infection. Here, we describe such a case involving an immunocompetent patient who developed sepsis and multiple organ dysfunction syndrome (MODS) following surgical evacuation of an intracerebral hemorrhage. The patient exhibited extensive multi-system involvement with an exceptionally high viral load. Through comprehensive management, including mechanical ventilation, continuous renal replacement therapy (CRRT), and IVIG administration, the patient’s consciousness gradually recovered, organ function improved significantly, and overall clinical status stabilized.

## Case presentation

The patient was a 51-year-old obese male teacher who was admitted to Tongde Hospital of Zhejiang Province on December 26, 2023, with a diagnosis of cerebellar hemorrhage. His medical history was significant for hypertension and type 2 diabetes mellitus. The patient had a history of poor adherence to dietary and exercise regimens, leading to suboptimal glycemic control. On admission, his hemoglobin A1c was markedly elevated at 12.8% (reference range: 3.6–6.5%). On emergency admission, his serum creatinine level was 140 μmol/L, although he denied any prior history of kidney disease. The patient underwent emergency surgery upon admission, which included robot-assisted neuroendoscopic craniotomy for resection of a right posterior inferior cerebellar artery (PICA) aneurysm, excision of a right cerebellar arteriovenous malformation (AVM), right-sided decompressive craniectomy, evacuation of the cerebellar hematoma, scalp flap reconstruction, and placement of a lumbar cistern drain for external drainage. Following the procedure, he was transferred to the intensive care unit (ICU) for postoperative management. Upon admission, the patient was in a state of mild coma, accompanied by fever with a body temperature of 38.5 °C. Complete blood count revealed a white blood cell count of 10 × 10^9^/L, with a neutrophil percentage of 81.9% and a lymphocyte count of 0.5 × 10^9^/L (CD4 + cells accounting for 49.2%). Hemoglobin level was 118 g/L, and platelet count was 133 × 10^9^/L. As his level of consciousness improved, he became able to follow commands and move spontaneously. One week later, after successful ventilator weaning, he was transferred to the neurosurgery ward, at which time his hemoglobin level was 95 g/L.

**Figure 1:**
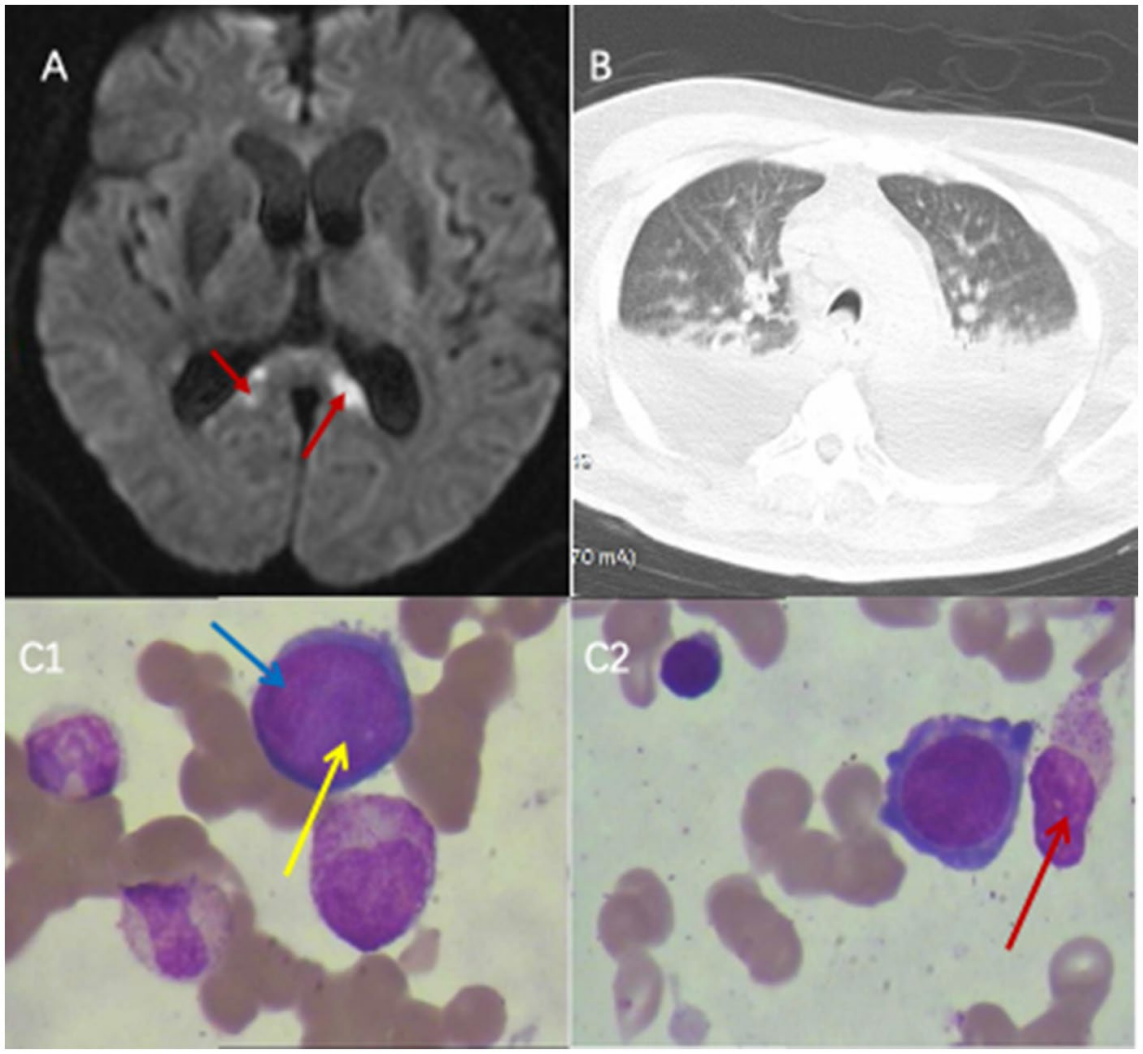
Results of head MRI, lung CT, and bone marrow puncture. **(A)** Brain magnetic resonance imaging (MRI) examination: Brain MRI revealed acute cerebral infarctions near the splenium of the corpus callosum and the posterior horns of the lateral ventricles. **(B)** Before being re-admitted to the ICU, the lung CT scan indicated bilateral pleural effusion. **(C1, C2)** Bone marrow smear of the patient. Proerythrocytes infected by parvovirus B19. Blue arrows indicated proerythroblasts, yellow arrows indicated viral inclusion bodies, red arrow indicates the plasma cells.

During his stay in the neurosurgery ward, the patient developed a progressive decline in hemoglobin levels (95 g/L → 58 g/L) (Table 1), accompanied by persistent fever and new-onset neurological symptoms, including speech disturbance and facial twitching. Lumbar puncture revealed an opening pressure of 310 mmH₂O. Cerebrospinal fluid (CSF) analysis showed a white blood cell count of 12 × 10^6^/L, with 70% lymphocytes. Biochemical examination of the CSF indicated a glucose level of 2.81 mmol/L, chloride concentration of 120.6 mmol/L, and protein concentration of 641 mg/L. Synchronous blood tests showed a glucose level of 7.14 mmol/L and chloride of 103.2 mmol/L. Brain MRI revealed acute cerebral infarctions near the splenium of the corpus callosum and the posterior horns of the lateral ventricles (Figure 1A). However, these imaging findings did not fully explain the patient’s worsening mental status and persistent fever. His condition continued to deteriorate, presenting with high-grade fever (up to 39.5 °C), lethargy, refractory anemia (hemoglobin decreased from 95 g/L to 58 g/L despite a negative stool occult blood test), hypoxemia (lowest SpO₂ = 85% on a reservoir mask), and generalized edema, necessitating urgent readmission to the ICU.

**Table 1:** Laboratory data.

Variable	Reference range	First ICU admission	ICU discharge	Readmission to discharge the ICU	D7 after ICU readmission	1 month after ICU readmission	2 months after ICU readmission	Final transfer out of ICU
Hemoglobin (g/dL)	130–175	118	95	59	60	62	51	56
Platelet count (10^9/L)	125–350	133	170	109	129	45	51	100
Redcell count (10^12/L)	4.3–5.8	3.75	3.22	2	2.08	2.09	1.6	1.74
Reticulocyte count (%)	0.5–1.5	/	/	0.14	0.15	1.06	5.78	2.22
White blood cell count (10^9/L)	3.5–9.5	10.0	14.2	8.9	5.9	3.5	6.3	6.5
Alkaline phosphatase (U/L)	30–120	53	69	94	231	526	984	517
Gamma-glutamyl transferase (U/L)	10–60	40	35	126	280	785	679	385
Total bilirubin (μmol/L)	0.0–26.0	11.4	15.2	12.2	9.1	28.1	26.3	14.4
Serum creatinine (mmol/L)	57–110	135	119	99	114 (underwent CRRT)	167	359	327

**Figure 2:**
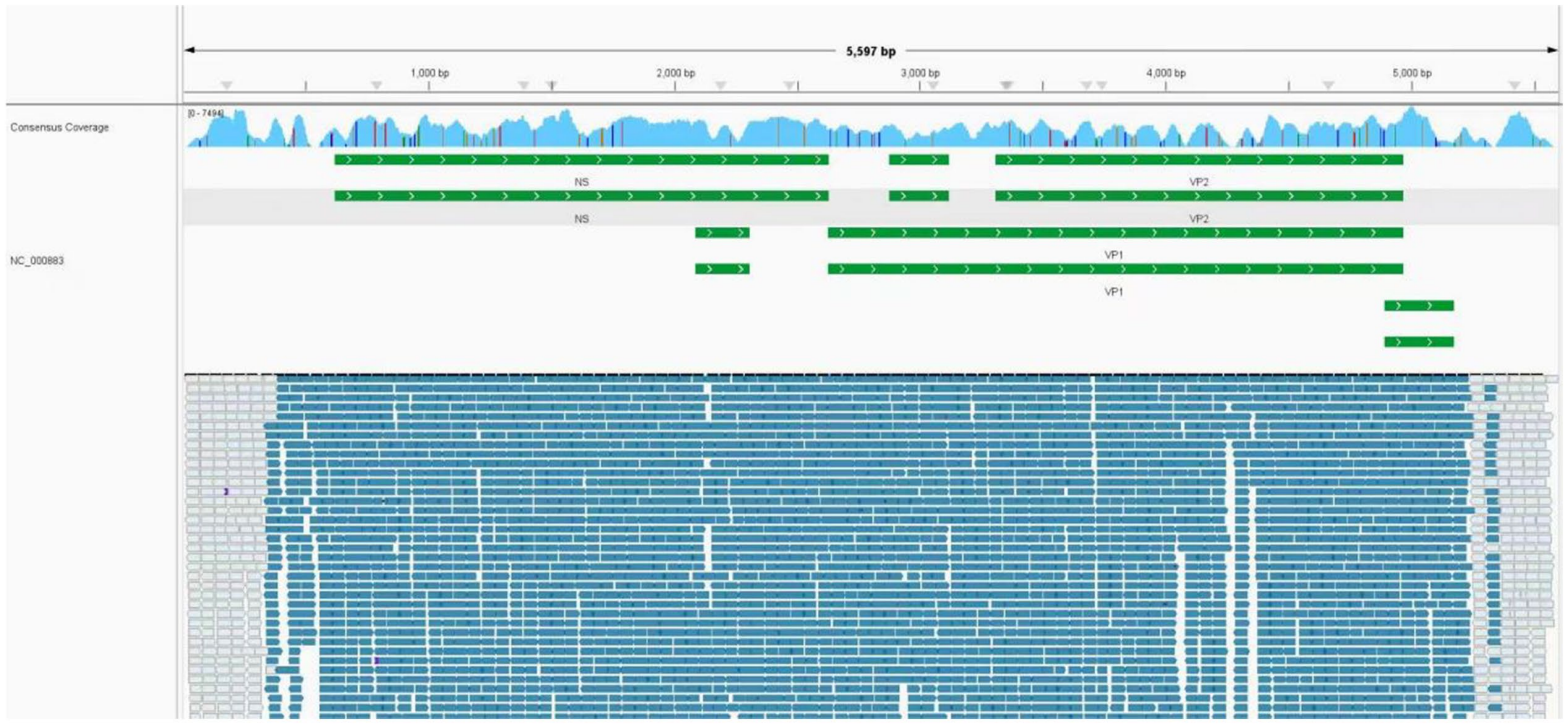
Results of next-generation sequencing in the peripheral blood. Mapping results of nucleotide sequences distributed along the genome of parvovirus B19 in the peripheral blood to parvovirus B19 reference genome NC_000883. The human parvovirus B19 genome has a total length of 5,594 base pairs (bp). The sequencing coverage spanned 5,593 out of 5,594 bp, achieving a coverage depth of 99.9821%.

Upon ICU readmission, the patient underwent endotracheal intubation and mechanical ventilation. Chest computed tomography revealed pulmonary infiltrates and bilateral pleural effusions (Figure 1B). Metagenomic next-generation sequencing detected high sequence counts of human PVB19 in the CSF, pleural fluid, and blood (Table 2; Figure 2). The patient developed persistent severe anemia, with a hemoglobin nadir of 50 g/L, accompanied by prolonged reticulocytopenia (reticulocyte percentage: 0.14–0.26%; absolute count: 0.18–0.53 × 10^12^/L) (Table 1) lasting approximately one week. Serum ferritin levels were markedly elevated (>1,500 ng/mL; reference range: 23.9–336 ng/mL). Bone marrow aspiration demonstrated markedly suppressed erythropoiesis with a predominance of proerythroblasts and early erythroblasts, consistent with PRCA (Figure 1C). Persistent lymphopenia (0.5 × 10^9^/L) indicated an immunosuppressed state. Given the immunodeficiency and high viral load, IVIG therapy was administered. Renal function progressively deteriorated, as evidenced by rising serum creatinine levels and oliguria, necessitating CRRT. Following treatment, PVB19 levels in both the CSF and blood declined significantly (Table 2; Figure 2). The patient regained consciousness, exhibiting improved responsiveness, resolution of facial twitching, and successful weaning from mechanical ventilation and renal replacement therapy. Normalization of reticulocyte counts prompted a transfer to nephrology for further management. The patient was discharged from the ICU on April 7, 2024.

**Table 2:** Test results of mNGS in body fluid samples of the patient.

Item specimen	The types of pathogens detected by mNGS	Viral DNA/RNA quantification (sequence count)
CSF1 (diagnosis)	PVB19	558,465
CSF2 (12 days after diagnosis)	PVB19	95
CSF3 (19 days after diagnosis)	PVB19	134
CSF4 (26 days after diagnosis)	PVB19	7
CSF5 (39 days after diagnosis)	CMV	56
CSF6 (3 month after diagnosis)	PVB19	4
Blood1 (1 day after diagnosis)	PVB19, Circovirus	1,074,518, 5
Blood2 (20 days after diagnosis)	PVB19, CMV	3, 39
Blood3 (30 days after diagnosis)	CMV, Circovirus, PVB19	1,511, 14, 8
Blood (3 month after diagnosis)	CMV, Circovirus, PVB19	20, 72, 25
Pleural effusion (1 day after diagnosis)	PVB19	29,658

CSF, cerebrospinal fluid; CMV, cytomegalovirus.

**Figure 3:**
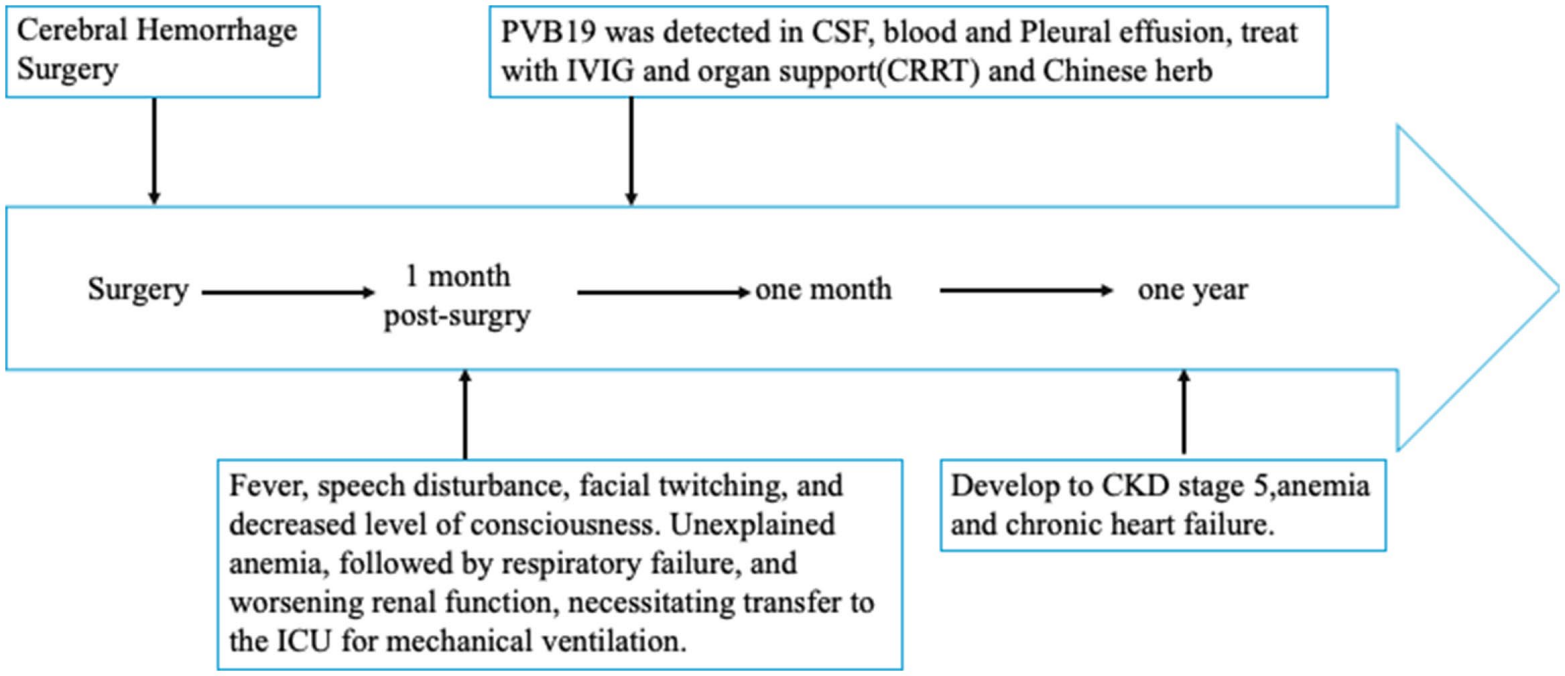
Timeline depicting the disease course of the patient. The timeline illustrates the different events in this patient’s treatment and disease progression and prognosis.

A one-year follow-up was conducted for the patient. Intermittent blood tests performed by the attending physician showed a gradual improvement in reticulocyte counts, while metagenomic next-generation sequencing of peripheral blood revealed only trace levels of parvovirus B19 sequences. Consequently, further immunoglobulin therapy was deemed unnecessary. The patient’s hemoglobin level progressively increased from 50 g/L to 65 g/L. However, renal function failed to recover, necessitating ongoing maintenance hemodialysis. In addition, cardiac function progressively deteriorated, resulting in multiple ICU readmissions for episodes of acute left ventricular failure. The timeline of the patient’s clinical course, treatment interventions, and outcomes is summarized in Figure 3.

## Discussion

PVB19 typically causes self-limited illnesses, such as erythema infectiosum in children and transient arthritis in healthy adults (3). However, in immunocompromised patients or those with chronic anemia, PVB19 infection can lead to severe or even fatal complications, including chronic PRCA and hydrops fetalis (4, 6, 13).

At first glance, the patient appeared to be a typical postoperative case of cerebral hemorrhage with no prior history of immunodeficiency. However, lymphocyte counts remained persistently below the normal reference range throughout the clinical course, suggesting an underlying state of immunosuppression. In light of the combined effects of major surgical trauma, acute intracerebral hemorrhage, poorly controlled type 2 diabetes mellitus, and possible underlying chronic kidney dysfunction, it is highly plausible that the patient was in a state of relative or subclinical immunosuppression—which is easily overlooked in routine clinical assessment yet sufficient to increase susceptibility to severe parvovirus B19 infection. In this patient, unexplained and subsequently persistent anemia developed during the hospitalization period on the neurosurgical ward following surgery for intracerebral hemorrhage. As far as we know, the patient had no history of prior blood transfusion, and no transfusion was administered during surgery, supporting that the profound anemia developed *de novo* in the postoperative period. Potential causes of anemia were systematically evaluated before attributing the condition to a rare infection such as parvovirus B19. Acute blood loss was excluded by negative fecal and gastric occult blood tests, unremarkable upper endoscopy, and abdominal CT scan. All of which yielded negative. Furthermore, laboratory investigations during the period of progressive hemoglobin decline showed normal total bilirubin and indirect bilirubin levels. Red blood cell indices—including mean corpuscular volume (MCV), mean corpuscular hemoglobin (MCH), and mean corpuscular hemoglobin concentration (MCHC)—were within reference ranges. Urinalysis was negative for bilirubin and urobilinogen, and serum levels of folate and vitamin B12 were normal. These results effectively rule out both acute hemorrhage and hemolytic anemia as etiologies of the patient’s anemia. Based on the patient’s clinical course and serial complete blood count (CBC) trends—detailed in Table 1—the hemoglobin level remained persistently low, while white blood cell and platelet counts gradually recovered during hospitalization. This dissociated cytopenia pattern (isolated anemia with recovery of other lineages) strongly argues against bone marrow failure syndromes such as aplastic anemia, myelodysplastic syndromes (MDS), or acute leukemia, which typically present with pancytopenia or multilineage dysplasia. Drug-induced marrow suppression was deemed unlikely given the absence of typical offending agents and the presence of isolated red cell aplasia. The combination of profound reticulocytopenia, bone marrow erythroid hypoplasia, and high PVB19 viral load across multiple compartments ultimately pointed to PVB19-associated pure red cell aplasia as the unifying diagnosis. Human parvovirus B19 (PVB19) primarily infects erythroid progenitor cells in the bone marrow, particularly colony-forming unit–erythroid (CFU-E) cells, which express the P antigen—the cellular receptor for viral entry (14). The viral capsid proteins VP1 and VP2 mediate binding to these cells, leading to cell lysis and transient suppression of erythropoiesis (15). This results in reticulocytopenia and, in some cases, severe anemia, especially in individuals with increased red cell turnover or impaired immunity. Bone marrow aspiration in this patient (Figure 1C) demonstrated marked erythroid hypoplasia, with only occasional proerythroblasts and early basophilic erythroblasts present—findings consistent with arrested erythroid maturation, consistent with PVB19-associated bone marrow suppression.

Infection with PVB19 can manifest with a variety of clinical conditions. The uniqueness of this case lies in the development of multiple organ dysfunction secondary to PVB19 infection involving the central nervous system (encephalitis/meningitis), hematopoietic system (pure red cell aplasia), and kidneys (acute kidney injury), along with hepatic dysfunction—manifestations that collectively highlight the systemic pathogenic potential of parvovirus B19 beyond its classic hematologic effects. Recent reports have described PVB19-associated encephalitis, meningitis, peripheral neuropathy, and even stroke-like presentations (14). The patient developed altered mental status and new-onset facial twitching during neurosurgical admission, temporally associated with PVB19 infection, raising the possibility of viral-induced neurological complications. The underlying mechanism may involve post-surgical disruption of the blood–brain barrier combined with virus-induced inflammatory responses, which increase barrier permeability and potentially permit direct viral invasion of the central nervous system (16, 17). Notably, routine CSF biochemical tests including cell count, protein, and glucose showed no typical infection-related abnormalities after surgery. Therefore, in postoperative patients who develop neurological deterioration but exhibit negative results on conventional CSF testing, atypical pathogen infections should be considered, and molecular diagnostic tools, such as metagenomic next-generation sequencing, should be employed promptly (18). The patient also developed impaired hepatic and renal function (Table 1). The mechanisms underlying both acute and chronic renal impairment in this case remain unclear but are likely multifactorial. Retrospective assessment revealed elevated baseline creatinine and proteinuria, suggesting underlying chronic kidney disease. Following PVB19 infection, the patient developed acute renal deterioration, progressing to end-stage kidney disease requiring maintenance hemodialysis. We hypothesize that this deterioration may be attributable to PVB19-induced structural kidney injury, potentially leading to collapsing glomerulopathy—a lesion increasingly reported in viral-associated nephropathies, including those linked to parvoviral infection (19). PVB19-induced endothelial injury triggering thrombotic microangiopathy (TMA) and impairing renal perfusion, and high-grade viremia activating a cytokine storm that exacerbates organ damage (20). Renal pathology following PVB19 infection may also involve deposition of circulating immune complexes, local in-situ immune complex formation, or both processes occurring simultaneously (21). Unfortunately, tissue biopsies were not performed due to clinical constraints and patient/family preference to preclude direct histological confirmation of parvovirus B19–mediated organ injury. The route of parvovirus B19 infection in this patient remains unclear. Previous reports have described fetal cases in which parvovirus B19–induced severe anemia was followed by intracranial hemorrhage after intrauterine transfusion (7). In contrast, in our adult patient, cerebellar hemorrhage clearly preceded the onset of anemia, and blood transfusion was administered only after anemia had developed—indicating that the hemorrhage was not a consequence of transfusion-related hemodynamic shifts. Given the high seroprevalence of parvovirus B19 in the general population (2), reactivation of latent virus or acquisition of new infection is plausible under conditions of impaired immunity. We hypothesize that during the acute phase of intracerebral hemorrhage, the patient’s physiological stress—combined with comorbidities such as hypertension and diabetes—led to a transient state of immunosuppression. This may have facilitated either reactivation of latent parvovirus B19 or primary infection from environmental exposure, followed by hematogenous dissemination and potential crossing of the compromised blood–brain barrier, thereby contributing to systemic and possibly central nervous system involvement.

The diagnosis of parvovirus B19 infection typically hinges on the detection of virus-specific IgM antibodies and/or viral DNA in serum, particularly PCR-based detection of viral DNA—is considered essential for definitive diagnosis in this population (22). In our case, neither serological testing for parvovirus B19 IgM nor targeted PCR assays were performed on blood, cerebrospinal fluid (CSF), or pleural fluid, primarily due to limited availability of these tests at our institution and an initial lack of clinical suspicion for parvovirus B19 infection. Notably, parvovirus B19 DNA was incidentally identified in blood, CSF, and pleural effusion samples through metagenomic next-generation sequencing (mNGS). While the presence of viral sequences by mNGS does not, by itself, confirm active infection or establish causality-given the possibility of latent carriage or environmental contamination. We interpreted these findings in the context of the patient’s evolving clinical course. Critically, serial mNGS analyses revealed a dynamic increase in parvovirus B19 read counts that paralleled clinical deterioration, followed by a decline in viral load coinciding with clinical improvement after empirical antiviral therapy. This temporal correlation strengthened the likelihood that parvovirus B19 was the etiologic agent rather than a bystander. Although the absence of confirmatory serology and targeted PCR represents a diagnostic limitation, it did not ultimately compromise clinical decision-making or patient outcomes. This case underscores both the potential utility and interpretive challenges of mNGS in diagnosing rare viral infections, particularly when conventional diagnostics are unavailable or inconclusive.

Currently, no specific antiviral therapy exists for PVB19 infection, and management primarily relies on immune modulation and supportive care. Existing guidelines do not provide definitive recommendations regarding the dosage or duration of IVIG therapy for PVB19. In patients with severe anemia, IVIG is commonly administered at 400 mg/kg/day for five consecutive days, providing neutralizing antibodies that may accelerate viral clearance (14). In this patient, IVIG treatment was extended to 21 days, during which serial monitoring demonstrated a marked reduction in viral load. The prolonged course was necessitated by persistent severe pure red cell aplasia and a subsequent hospital-acquired, drug-resistant bacterial infection. Although the optimal timing and duration of IVIG therapy remain controversial, a prolonged regimen may be warranted in patients with severe anemia complicated by multiple organ failure. For patients presenting with unexplained anemia following cerebral hemorrhage surgery, postoperative differential diagnosis should include atypical pathogens, such as PVB19, particularly when conventional antimicrobial therapy is ineffective and major sources of bleeding have been excluded. Metagenomic next-generation sequencing (23) is a valuable tool for identifying complex infections and can substantially reduce the time to diagnosis.

## Conclusion

This case highlights the rare but potentially severe clinical manifestations of PVB19 infection following cerebral hemorrhage surgery, particularly in patients presenting with unexplained anemia. Accurate diagnosis requires clinician vigilance and the use of advanced molecular testing techniques, while effective management depends on a comprehensive approach, including IVIG therapy and supportive care to preserve multi-organ function. This report offers valuable insights into the diagnosis and management of severe anemia in postoperative patients, emphasizing the importance of broadening differential diagnoses in cases of unexplained anemia with concomitant multi-organ dysfunction. Early recognition of atypical infections may improve patient outcomes.

## Data Availability

The original contributions presented in the study are included in the article/supplementary material, further inquiries can be directed to the corresponding author/s.
